# Chromogranin-A Expression as a Novel Biomarker for Early Diagnosis of Colon Cancer Patients

**DOI:** 10.3390/ijms20122919

**Published:** 2019-06-14

**Authors:** Xueli Zhang, Hong Zhang, Bairong Shen, Xiao-Feng Sun

**Affiliations:** 1 School of Medicine, Institute of Medical Sciences, Örebro University, SE-70182 Örebro, Sweden; zhang.xueli@oru.se; 2 Centre for Systems Biology, Soochow University, Suzhou 215006, China; 3 Department of Oncology and Clinical and Experimental Medicine, Linköping University, SE-58183 Linköping, Sweden

**Keywords:** CHGA, colon cancer, biomarker, early diagnosis, logistic regression, meta-analysis, PPI

## Abstract

Colon cancer is one of the major causes of cancer death worldwide. The five-year survival rate for the early-stage patients is more than 90%, and only around 10% for the later stages. Moreover, half of the colon cancer patients have been clinically diagnosed at the later stages. It is; therefore, of importance to enhance the ability for the early diagnosis of colon cancer. Taking advantages from our previous studies, there are several potential biomarkers which have been associated with the early diagnosis of the colon cancer. In order to investigate these early diagnostic biomarkers for colon cancer, human chromogranin-A (CHGA) was further analyzed among the most powerful diagnostic biomarkers. In this study, we used a logistic regression-based meta-analysis to clarify associations of CHGA expression with colon cancer diagnosis. Both healthy populations and the normal mucosa from the colon cancer patients were selected as the double normal controls. The results showed decreased expression of CHGA in the early stages of colon cancer as compared to the normal controls. The decline of CHGA expression in the early stages of colon cancer is probably a new diagnostic biomarker for colon cancer diagnosis with high predicting possibility and verification performance. We have also compared the diagnostic powers of CHGA expression with the typical oncogene KRAS, classic tumor suppressor TP53, and well-known cellular proliferation index MKI67, and the CHGA showed stronger ability to predict early diagnosis for colon cancer than these other cancer biomarkers. In the protein–protein interaction (PPI) network, CHGA was revealed to share some common pathways with KRAS and TP53. CHGA might be considered as a novel, promising, and powerful biomarker for early diagnosis of colon cancer.

## 1. Introduction

Colon cancer is one of the leading causes of cancer death worldwide [1]. In 2018, there were more than 1,096,601 new diagnosed colon cancer cases, and around 551,269 patients were dead from the colon cancer worldwide [2]. The advanced modern medicines and surgery techniques have nowadays benefited the stage I and II colon cancer patients and their five-year survival rate has reached to 90% [3]. However, the five-year survival rate for the stage IV colon cancer patients is only around 10% [3]. Moreover, more than 50% of colon cancer patients are already at the late stages when they are clinically diagnosed [4]. As such, it is urgent to find more convenient and accurate biomarkers for early diagnosis of colon cancer.

Although colonoscopy and biopsy pathological examination have been considered as the golden test for colon cancer final diagnosis and primary treatment [5], there are a number of early colon cancer cases which do not reveal the typical characteristics, making it difficult to make a clear diagnosis. Accumulating studies on the biomarkers in colon cancer diagnosis have partially solved the problem [6]. However, it is necessary to find more precise biomarkers which can complement the golden test for the early diagnosis of colon cancer.

Biomarkers as biological indicators and conditions have been widely believed to improve the diagnosis, therapeutic response, and prognosis of colon cancer [5]. Thus, many researcher groups, including ours, have been putting a lot of effort into identifying the significance of various biomarkers for colon cancer [7,8,9,10]. In our previous work, we established an integrated biomarker database for colorectal cancer (CRC): CBD (http://sysbio.suda.edu.cn/CBD/index.html), in which we collected and summarized the ontology-based biomedicine information from all the published CRC biomarkers up to 2018 [11]. In the CBD, we recorded 62 biomarkers concerning the diagnosis for early-stage colon cancer. However, none of such biomarkers have been identified as ideal clinical applications. Hence, it is markedly important to further explore new promising biomarkers concerning early diagnosis, therapeutic response, and prognosis for CRC.

Human chromogranin-A (CHGA), as a 439-residue-long protein in the secretory granules of many normal and neoplastic neuroendocrine cells, plays a major role in the protein co-stored and co-released with catecholamines from secretory vesicles in adrenal medulla and postganglionic sympathetic axons [12,13]. Expression of CHGA has been proven to be associated with prognosis in CRC [14,15,16], and has been currently accepted as the main biomarker for neuroendocrine neoplasms [17,18]. Recently, CHGA has been approved as an early diagnosis biomarker for gastric cancer [19], prostate cancer [20], and pancreatic neuroendocrine tumors [21]. However, there is no study concerning CHGA expression and early diagnosis in colon cancer.

KRAS as a typical oncogene for cancer initiation and progression has been proven in many cancer types, including CRC, with strong impact on early diagnosis, cancer progression, and prognosis [22]. There are up to 50% of CRC patients with KRAS gene mutations in their early stages, and half of such patients are unlikely to benefit from the antibody therapy [22]. TP53 as a classic tumor suppressor has revealed more than 60% of CRC patients with TP53 mutations, which is one of the commonest genetic events in the development of human CRC [23]. The MKI67 is a biomarker of cellular proliferation and has been reported as an independent index for CRC cell growth [24]. The expression of MKI67 has been gradually increased from normal tissue, adenomas to adenocarcinomas, in CRC patients. The overexpression of MKI67 has been considered as the significant biomarker and predictor for primary CRC screening [25].

Machine learning has been widely utilized in bioinformatics for screening potential biomarker candidates and their complex molecular networks [26]. The protein–protein interaction (PPI) network has been proven with high confidence in biomedicine research [27]. In a previous study, we utilized the support vector machine (SVM) and regression tree to predict new potential colon cancer biomarker based on the PPI network, with high indication that CHGA expression, among several others, could be a promising significant biomarker for early diagnosis of colon cancer [28]. To our knowledge, there is no report concerning the association of the CHGA with early diagnosis of colon cancer, although it is necessary to be further verified.

In this study, we have used logistic regression-based meta-analysis, a quantitative review method to detect the diagnostic value of different biomarkers in medical researches [29], and a huge number of gene expression (GE) data for different populations from the Gene Expression Omnibus (GEO) database, as a well-known biomedicine database, to investigate the diagnostic value of CHGA expression as a biomarker, compared to the healthy populations and the normal mucosa from the colon cancer patients. We showed that CHGA expression might be considered as a novel biomarker for early diagnosis of colon cancer, as compared to most well-studied expressions of KRAS, TP53, and MKI67.

## 2. Results

### 2.1. Logistic Regression-Based Meta-Analysis Has Been Used in This Study

**Figure 1:**
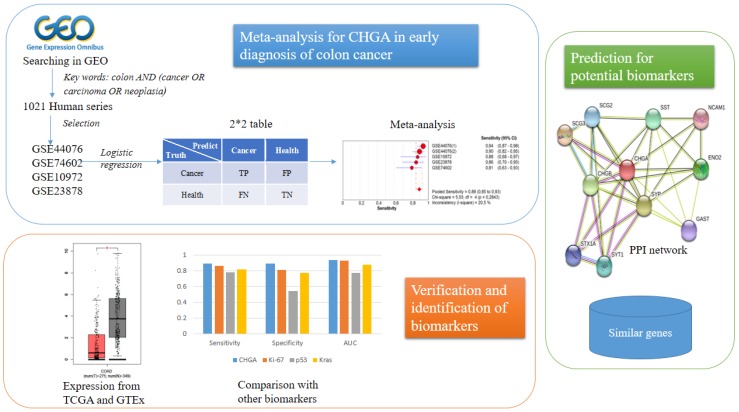
Study pipeline. In this study we used the human microarray Gene expression (GE) data from the Gene Expression Omnibus (GEO) database and logistic regression to formulate the 2 × 2 table for meta-analysis. After the chromogranin-A (CHGA) diagnostic meta-analysis, we utilized the RNA-seq data from The Cancer Genome Atlas (TCGA) and Genotype-Tissue Expression (GTEx) to test the CHGA expression in colon cancer patients and healthy controls to verify the results for meta-analysis. Meanwhile the diagnostic meta-analysis for several other reported biomarkers were also conducted using the same datasets. Finally, we predicted several CHGA-associated biomarkers from the protein-protein interaction (PPI) network and GE level.

The Study Design Was Represented in Figure 1.

### 2.2. Data Collection

Microarray human series were collected from the GEO database using a keywords search. There were 1021 GEO datasets collected and 1012 were excluded due to lack of stage information. There were two datasets without detailed cancer patients and experiments information. One dataset did not contain enough samples and two datasets did not include the GE data for CHGA. Eventually, there were four ideal datasets (GSE44076, GSE74602, GSE10972, and GSE23878), including 187 colon cancer patients and 226 normal controls for the analyses. Table 1 shows the characteristics of the included studies.

### 2.3. Logistic Regression

Logistic regression was utilized to clarify the expression data to establish the 2 × 2 table for meta-analysis (Table 2). GSE44076 contains 2 groups of control: one from healthy samples, the other one from corresponding normal adjacent mucosa of patients.

### 2.4. CHGA in the Diagnosis of Early-Stage Colon Cancer

**Figure 2:**
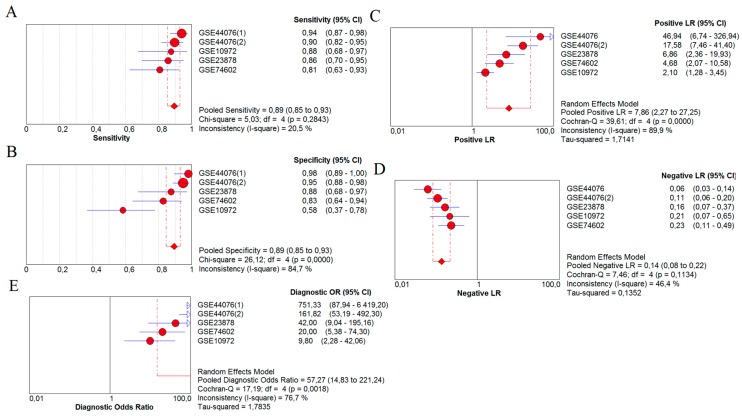
Forest plots of meta-analysis for CHGA as diagnostic biomarker in early-stage colon cancer. Different points represented various studies in the meta-analysis, which were arranged from high to low by effect size. (**A**) forest plot of sensitivity (0.89). By calculating the I^2^ (20.5%), no significant heterogeneity was found for this meta-analysis; (**B**) forest plot of specificity (0.89); (**C**) forest plot of positive-likelihood ratio (PLR) (7.86); (**D**) forest plot of negative-likelihood ratio (NLR) (0.14); (**E**) forest plot of diagnostic odds ratio (DOR) (57.27).

Figure 2 presents the results, using forest plots, from the meta-analysis. Figure 2A shows the sensitivity of the forest plot for CHGA as a biomarker in the diagnosis of early-stage colon cancer, from which a pooled sensitivity of 0.89 (0.85 to 0.93) was calculated. The specificity forest plot was drawn in Figure 2B (0.89, 0.85 to 0.93). Figure 2C,D present the likelihood ratios for CHGA: positive-likelihood ratio (PLR) 7.86 (2.27 to 27.25) and negative-likelihood ratio (NLR) 0.14 (0.08 to 0.22). CHGA performed a diagnostic odds ratio (DOR) of 57.27 (14.83 to 222.24), which is available on the forest plot in Figure 2E. All these statistics were based on 95% confidence.

**Figure 3:**
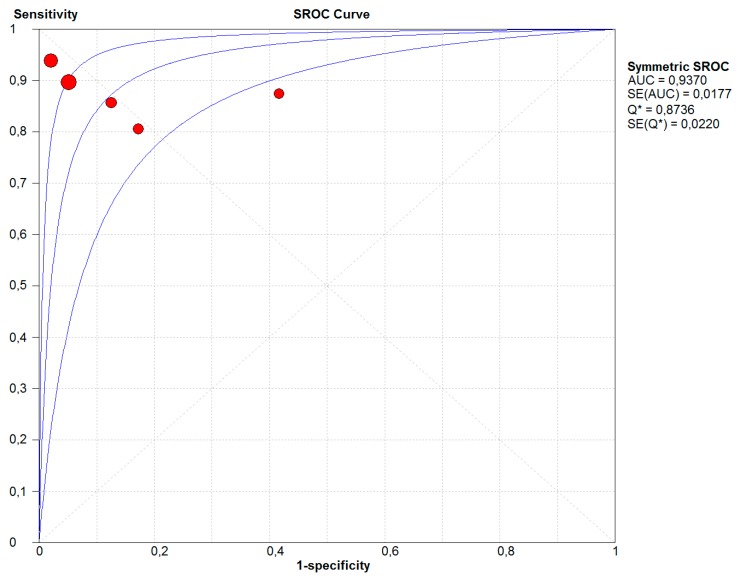
Summary receiver operator characteristic (SROC) curve for CHGA as an early diagnostic biomarker in colon cancer. Different points represent different studies in the meta-analysis and the size of points is the number of patients. There are three lines on the SROC curve: The middle line is the SROC curve fitted by the sensitivity (y-axis) and 1-specificity (x-axis) for corresponding studies, and the other two lines are the confidence interval. The SROC curve reflects diagnostic accuracy for biomarkers and the bigger size of area under curve (AUC) presents the better diagnostics accuracy for biomarkers. CHGA shows high diagnostic accuracy (AUC = 0.9370) in colon cancer.

The summary receiver operator characteristic (SROC) curve for CHGA diagnostic meta-analysis is presented in Figure 3 and CHGA showed high diagnostic accuracy: area under curve (AUC) 0.9370 and Q value 0.8736.

### 2.5. Comparison of the CHGA with Other Biomarkers

In order to make a comparison for the diagnostic effect of the CHGA with the identified biomarkers, the diagnostic meta-analysis for MKI67, TP53, and KRAS were conducted using the same datasets. The results for these meta-analyses are listed in Table 3.

### 2.6. Verification in RNA-Seq Data

**Figure 4:**
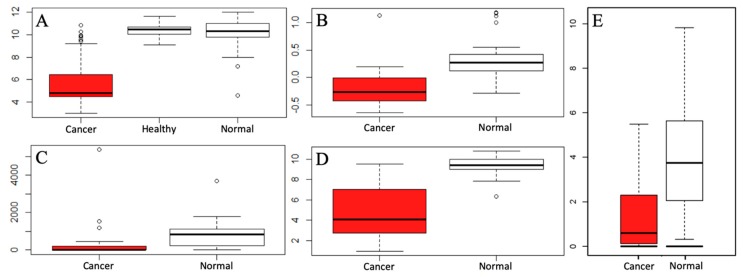
CHGA expression for colon cancer (in red solid box) and normal controls (in empty box) in microarray and RNA-seq data. (**A**) CHGA expression for colon cancer patients, healthy control, and normal adjacent mucosa of the colon cancer patients from the GSE44076 dataset; (**B**) CHGA expression for colon cancer patients and normal adjacent mucosa of the colon cancer patients from the GSE74602 dataset; (**C**) CHGA expression for colon cancer patients and normal adjacent mucosa of the colon cancer patients from the GSE10972 dataset; (**D**) CHGA expression for colon cancer patients and normal control of adjacent mucosa of the colon cancer patients from the GSE23878 dataset; (**E**) CHGA expression in colon cancer patients and normal controls from RNA-seq data from TCGA and GTEx.

The CHGA expression of the microarray datasets was verified in the RNA-seq data from GTEx and TCGA databases. Figure 4A–D presents the expression of the microarray datasets from our meta-analysis. Figure 4E shows the CHGA expression levels for colon cancer patients and healthy controls in TCGA and GTEX databases. The expression of the CHGA levels was markedly reduced in the colon cancer patients as compared with the CHGA expression in the normal controls in both microarray and RNA-seq data.

### 2.7. CHGA-Related PPI Networks and Biological Explanation

**Figure 5:**
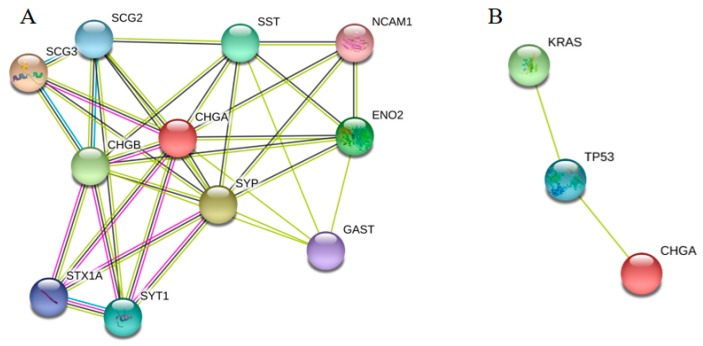
Closest PPI network for CHGA (**A**). The String database was utilized to draw the human PPI networks showing associations of CHGA with its closest neighbors. Relationships of CHGA, TP53, and KRAS (**B**). Several well-known biomarkers such as KRAS, TP53, and MKI67 were input together with CHGA in the String database, showing that TP53 had interaction relationships with CHGA and KRAS. Different points represented different proteins and different lines indicated the interactions from different evidences. Green lines: The evidence from neighborhood genes; Black line: The proteins co-expression; Purple line: The evidence from the experiment; Blue line: The evidence from curated databases.

Our previous study showed that CRC biomarkers had strong relationships on the PPI network [10]. Therefore, we supposed that the close neighbors with CHGA on the PPI network had high possibility to be further biomarkers. In order to further analyze the biological interactions for CHGA, PPI networks were constructed for both its closest proteins and other biomarkers in Figure 5. SCG3, SCG2, SST, NCAM1, ENO2, GAST, SYP, SYT1, STX1A, and CHGB, as the nearby proteins of the CHGA, were predicted as potential future early diagnostic biomarkers for colon cancer (Figure 5A). CHGA expression was found to be associated with KRAS and TP53 (Figure 5B).

In order to investigate the biological explanation for the reason why CHGA performed so well in the diagnosis of early-stage colon cancer, gene ontology (GO) analyses were conducted for the CHGA-related genes (from closest PPI and CHGA–TP53–KRAS PPI, Table 4). We found that CHGA and its closest genes were strongly associated with the regulations of cell communication and signaling at the biological function level (Table 4A). At the cellular component level, they are closely linked to the transport functions (Table 4B). Additionally, we found that CHGA and its related biomarkers (TP53 and KRAS) were mapped on the regulation of neuron death and cell death pathways (Table 4C).

### 2.8. Prediction for CHGA-Related Biomarkers from Expression Levels

**Figure 6:**
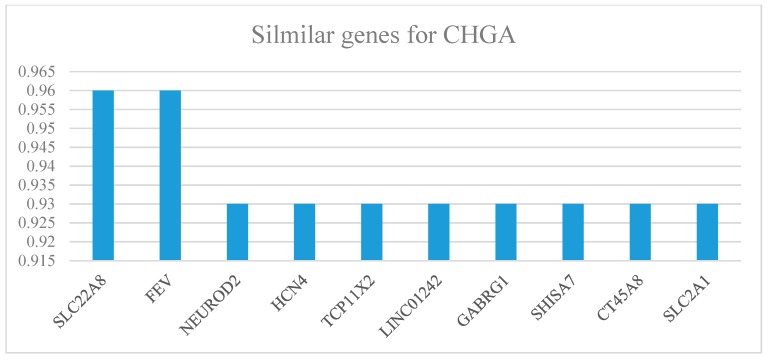
Similar genes that have similar expression patterns, ranked by Pearson correlation coefficients, for CHGA in colon cancer patients.

The genes with similar expression levels always performed relatively similar functions [30]. Figure 6 shows the similar expressions of the genes to CHGA in colon cancer, which were predicted as further biomarker candidates.

## 3. Discussion

Accumulating evidence has shown that the majority of colon cancer patients in their early stages (I and II) will benefit from modern cancer therapies, and their five-year survival rate has reached up to more than 90%. However, the five-year survival rate for the later stages (III and IV) of colon cancer patients remain at about 10% [3]. Moreover, more than 50% of colon cancer patients are already at the late stages when their cancer is clinically diagnosed [4]. The strict rule is that the earlier the diagnosis for cancers, including colon cancer, the better therapies the cancer patients will receive and the better the outcome the patients will have. It; therefore, appears significantly important to search for more convenient, accurate, and powerful biomarkers to meet such an urgent need for the early diagnosis of colon cancer.

Biomarkers as biological indicators and conditions have been widely shown to improve the diagnosis, therapeutic response, and prognosis of colon cancer [5]. Many cancer researchers, including our CRC research group, have been working on identifying the significance of various biomarkers for CRC [7,8,9,10]. In order to study the essential roles and important functions of biomarkers in CRC, we have created a CRC biomarker database [11], and have further analyzed, with AI-assisted verification, the potential applications of DNA, RNA, and protein biomarkers in diagnosis, therapy, and prognosis for CRC [10].

CHGA is a 439-Kd protein in the secretory granules of many normal and neoplastic neuroendocrine cells and it plays an essential role in the mechanisms of protein storage and release [13]. CHGA was considered as a biomarker for neuroendocrine neoplasms [17,18] and was approved, with microarrays and tissue arrays, as a potential biomarker for early cancer diagnosis of gastric cancer [19] and prostate cancer [20]. The majority of pancreatic neuroendocrine tumors showed CHGA positive immunostaining, and primary tumors with metastases revealed significantly less CHGA protein expression than primary tumors without metastases [21]. However, there is no study concerning CHGA in early diagnosis of colon cancer.

In this study, CHGA expression was found to be decreased in the early stages of colon cancer in patients, as compared to CHGA expression levels in both the healthy populations and the normal colon cancer mucosa from the colon cancer patients. This evidence indicated that CHGA might play an essential role in the initiation of colon cancer, from the normal colon tissue to early cancer. The down-expression of CHGA might be one of the critical mechanisms leading to colon cancer formation.

In one of our ongoing studies, CHGA expression was predicted as a promising early diagnosis biomarker for colon cancer via SVM and regression tree analysis, from the reported colon cancer diagnostic biomarkers’ topology features on the PPI network [28], and the diagnostic value of CHGA expression in colon cancer was further verified. Meta-analysis, with its high scientific confidence, has been used to detect the diagnostic value of different biomarkers in various diseases [31,32]. The GEO database has been widely used in bioinformatics analysis because it is a comprehensive database storing big amounts of GE data. As such, more and more studies have been recently focused on the meta-analysis based on the GEO datasets, and a majority of the meta-analysis began with GE differential analyses [33,34]. Our results showed even higher confidence than the GE differential analyses, since we predicted CHGA expression as a candidate biomarker by machine learning (SVM and regression tree) based on the published biomarkers [28].

According to our previous study, CRC biomarkers always had strong relationships on the PPI networks [10]. Hence, we predicted several valuable biomarkers from the CHGA closest PPI network (Figure 5A) which deserved further verification. In order to detect the functions and applications of these closest neighbors of the CHGA, literature verifications were performed. We searched relevant published papers from PubMed and Google scholar concerning these nearby proteins and cancers, and found that SCG3 was convinced as a prognostic biomarker for lung cancer [35] and rectal cancer [36]; SST expression was reported to have a strong relationship with advanced CRC [37]; NCAM1 was correlated with several human cancers [38]; ENO2 were predicted as treatment biomarker for acute lymphoblastic leukemia [39]; SYT1 expression was a candidate colon cancer biomarker [40]; STAX1 was proved to be associated with the transformation of high-grade tumors in the bladder cancer [41]; and as a paralog for CHGA, CHGB was a prognostic marker for the pancreatic neuroendocrine tumors [21]. Expression of SLC22A8 was considered as a valuable biomarker for the lung cancer treatment [42]. The NEUROD2 gene was related to the metastasis and the survival for colon cancer [43]. The SLC2A1 gene was related to the metabolic shift of colon cancer [44], and the HCN4 gene was correlated with low survival rate of multiple cancers [45].

A variety of biomarkers, as biological indicators in their pathways, have been widely used to improve the cancer diagnosis, therapeutic response, and prognosis, including for colon cancer [6]. A majority of cancer researchers, including our CRC research group, have been focusing on the typical oncogenes, such as KRAS [22], and classic tumor suppressors like TP53 [23], to clarify their significance of various biomarkers in colon cancer. KRAS, as a typical oncogene to initiate cancer development, has been proven in many cancer types, especially in CRC, with strong early diagnosis impact, even in the cancer progression and prognosis [22]. There are more than 50% of CRC patients with KRAS gene mutations in their early stages, and half of such patient are not benefiting from antibody therapy [22]. TP53, as a classic tumor suppressor, has revealed more than 60% of CRC patients with TP53 mutations, which is one of the commonest genetic events in the development of human CRC [23]. MKI67 is a biomarker of cellular proliferation and has been reported as an independent index for CRC cell growth [22]. The expression of MKI67 has been gradually increased from normal tissue, adenomas to adenocarcinomas, in CRC patients. The overexpression of MKI67 has been considered as the significant biomarker and predictor for primary CRC screening [25].

Majority of the candidate biomarkers predicted based on CHGA PPI and CHGA similar genes were further verified through the literatures studies. We showed that the candidate biomarkers were associated with the diagnosis, therapy, or prognosis of human cancers, including colon cancer, in which provided further evidence for the CHGA as a potential hub biomarker for the colon cancer. Furthermore, we found several important pathways for the CHGA and its closest PPI neighbors, as well as other biomarkers from GO annotation: The regulation of cell communication and signaling at the biological function level, the transport function, and the regulation of neuron death and cell death, which should be important for future colon cancer biomarker discovery. In the near future, there will be more practical machine learning techniques that will be introduced to the field of precise biomarker discovery, for the identification and verification of potential biomarkers for early diagnosis, therapy response, and prognosis in cancers.

## 4. Materials and Methods

### 4.1. Data Collection, Extraction, and Normalization

We followed the ‘‘Preferred Reporting Items for Systematic Reviews and Meta-Analyses’’ guidelines to conduct this meta-analysis.

Two investigators independently searched the GEO database for relevant studies up to February 2019. The following search terms were utilized: colon AND (cancer OR carcinoma OR neoplasia). We recorded the homo sapiens relevant series as a further filter.

The criteria to select the needed studies was as following: (1) The study contains the GE data for early-stage colon cancer patients and normal healthy controls. (2) There are clear descriptions for the patients’ situation and experiment methods. The datasets recorded the country, number, and source of included samples, and the experiment methods and platform were clearly introduced, for example: “Expression profiling by array,” platform “GPL13667.” (3) The suitable dataset should contain at least 30 samples. (4) The dataset should include the GE data for CHGA. R package “GEOquery” was used to download the GE data from the GEO database, and the data were standardized to log scale. The function “normalizeQuantiles” in “Limma” R package was utilized to further normalize the log-scaled GE data.

### 4.2. Logistic Regression and Diagnostic Meta-Analysis

We extracted the GE data of CHGA for both the cancer group and the control group, and implemented logistic regression analysis to get true positive (TP), false positive (FP), true negative (TN), and false negative (FN) results for diagnostic meta-analysis. In this logistic regression, GE level was used as the variable.

Diagnostic meta-analysis for early-stage colon cancer was conducted, and a random effects model was selected as a standard statistical method in this study, which did not need new data to train the models and allowed the differences among the different studies.

The statistical data were as follows: sensitivity, specificity, PLR, NLR, and DOR with corresponding 95% confidence intervals. The SROC curves were plotted based on the sensitivity and specificity. A random effect model was used for the statistics. The heterogeneity among studies was assessed by I² on sensitivity of the CHGA diagnostic test. I² < 50% was considered as small heterogeneity.

### 4.3. Verification Test

In order to verify the meta-analysis result, we used RNA-seq data from the TCGA and GTEx database to compare the CHGA expression level between colon cancer patients with healthy samples. Meanwhile we also drew the boxplot for CHGA expression in our collected microarray data.

### 4.4. CHGA PPI Network Construction, Biological Function Analysis, and Detection for CHGA Similar Genes

We drew the PPI network for searching for the neighbors for CHGA based on protein interaction evidence, and investigated the PPI relationships of CHGA with some reported biomarkers (TP53, KRAS, and MKI67). GO annotation was conducted for analyzing the biological functions of the CHGA-related genes and biomarkers. The genes with similar expression were considered as similar genes, and we used Pearson correlation coefficients to calculate the similar genes for CHGA to provide evidence for further prediction of new biomarkers.

### 4.5. Software and Tools

R language was used to download the GEO data and make normalization, and logistic regression was conducted by SPSS 22.0. Diagnostic meta-analysis and heterogeneity analysis were implemented by MetaDisc 1.4. Verification test and similar genes detection were executed on the Gene Expression Profiling Interactive Analysis (GEPIA) database. The String database was used to draw the PPI network and conduct the biological function analyses.

## 5. Conclusions

A logistic regression-based meta-analysis was used to analyze the significant roles and functions of CHGA expression as a novel and promising significant biomarker for early diagnosis of colon cancer patients, and to compare CHGA in early diagnosis with the well-known oncogene, KRAS, tumor suppressor, TP53, and cellular proliferative factor, MKI67, in colon cancer. CHGA might be a further biomarker for early colon cancer patients. Several other biomarkers from the PPI network, and similar genes to CHGA, were further predicted for future early diagnosis of colon cancer.

## Figures and Tables

**Table 1:** Characteristics of studies of primary colon cancers (PCC) included in the meta-analysis.

Datasets	Sample Number	Stage	Region	Source	Expression	Platform	PMID*
GSE44076	98/148	PCC	Barcelona	Tissue	Array	GPL13667	25215506
GSE74602	30/30	PCC	Singapore	Tissue	Array	GPL6104	NA
GSE10972	24/24	PCC	Singapore	Cell	Array	GPL6104	18538736
GSE23878	35/24	PCC	Riydh	Tissue	Array	GPL570	21281787

* Sample number: Patient/Control; PMID: PubMed ID.

**Table 2:** Logistic regression results (2 × 2 table).

Datasets	TP	FP	FN	TN*
GSE44076 (1)	92	1	6	49
GSE44076 (2)	87	5	10	93
GSE74602	25	5	6	24
GSE10972	21	10	3	14
GSE23878	30	3	5	21

* TP: True positive; FP: False positive; FN: False negative; TN: True negative.

**Table 3:** Comparison of CHGA with several typically identified biomarkers.

Biomarker	Sensitivity	Specificity	PLR	NLR	DOR	AUC	Q Value	I^2^*
CHGA	0.89	0.89	46.94	0.14	57.27	0.9370	0.8736	0.205
MKI67	0.86	0.81	4.60	0.18	27.65	0.9270	0.8615	0.527
TP53	0.78	0.54	1.81	0.64	2.36	0.7732	0.7129	0.78
KRAS	0.82	0.77	2.83	0.30	9.78	0.8745	0.8050	0.812

* Q value: the point closest to the ideal top left-hand concer (sensitivity = specificity) on SROC curve; I^2^: the measure for heterogeneity in meta-analysis.

**Table 4:** Biological function analysis results for CHGA-related genes and biomarkers. (**A**) Biological function (GO) for CHGA closest genes. (**B**) GO for CHGA closest genes. (**C**) GO for CHGA and TP53, KRAS.

(**A**)				
**GO ID**	**Pathway**	**Count**	FDR*	**Matching Genes**
GO:0010646	regulation of cell communication	9	0.0018	CHGA, CHGB, GAST, NCAM1, SCG2, SST, STX1A, SYP, SYT1
GO:0023051	regulation of signaling	9	0.0018	CHGA, CHGB, GAST, NCAM1, SCG2, SST, STX1A, SYP, SYT1
GO:0050433	regulation of catecholamine secretion	3	0.0018	CHGA, STX1A, SYT1
GO:0048583	regulation of response to stimulus	9	0.0022	CHGA, CHGB, GAST, NCAM1, SCG2, SST, STX1A, SYP, SYT1
GO:0009966	regulation of signal transduction	8	0.0028	CHGA, CHGB, GAST, NCAM1, SCG2, SST, STX1A, SYP
GO:0032940	secretion by cell	5	0.0064	CHGA, SCG2, SCG3, STX1A, SYT1
GO:0070887	cellular response to chemical stimulus	7	0.0083	CHGA, NCAM1, SCG2, SST, STX1A, SYP, SYT1
GO:0010469	regulation of signaling receptor activity	4	0.0084	CHGB, GAST, SCG2, SST
GO:0042221	response to chemical	8	0.0127	CHGA, GAST, NCAM1, SCG2, SST, STX1A, SYP, SYT1
GO:0045055	regulated exocytosis	4	0.014	CHGA, SCG3, STX1A, SYT1
(**B**)				
**GO ID**	**Pathway**	**Count**	FDR*	**Matching Genes**
GO:0030658	transport vesicle membrane	5	4.82 × 10⁻^6^	CHGA, SCG3, STX1A, SYP, SYT1
GO:0099503	secretory vesicle	7	1.33 × 10⁻^5^	CHGA, CHGB, SCG2, SCG3, STX1A, SYP, SYT1
GO:0030141	secretory granule	6	8.41 × 10⁻^5^	CHGA, CHGB, SCG2, SCG3, STX1A, SYT1
GO:0005576	extracellular region	8	0.00024	CHGA, CHGB, GAST, NCAM1, SCG2, SCG3, SST, STX1A
GO:0042583	chromaffin granule	2	0.00024	CHGA, SYT1
GO:0098588	bounding membrane of organelle	6	0.0019	CHGA, NCAM1, SCG3, STX1A, SYP, SYT1
GO:0012505	endomembrane system	8	0.003	CHGA, CHGB, NCAM1, SCG2, SCG3, STX1A, SYP, SYT1
GO:0005615	extracellular space	4	0.0125	CHGA, GAST, SCG2, SST
(**C**)				
**GO ID**	**Pathway**	**Count**	FDR*	**Matching Genes**
GO:1901214	regulation of neuron death	3	0.0023	CHGA, TP53, KRAS
GO:0060548	negative regulation of cell death	3	0.0449	CHGA, TP53, KRAS

*FDR: False discovery rate.
